# Identification of Dish-Based Dietary Patterns for Breakfast, Lunch, and Dinner and Their Diet Quality in Japanese Adults

**DOI:** 10.3390/nu13010067

**Published:** 2020-12-28

**Authors:** Nana Shinozaki, Kentaro Murakami, Keiko Asakura, Shizuko Masayasu, Satoshi Sasaki

**Affiliations:** 1 Department of Social and Preventive Epidemiology, School of Public Health, The University of Tokyo, 7-3-1 Hongo, Bunkyo-ku, Tokyo 113-0033, Japan; nana-s@m.u-tokyo.ac.jp (N.S.); stssasak@m.u-tokyo.ac.jp (S.S.); 2 Department of Environmental and Occupational Health, School of Medicine, Toho University, 5-21-16 Omori-Nishi, Ota-ku, Tokyo 143-8540, Japan; keiko.asakura@med.toho-u.ac.jp; 3 Ikurien-naka, 3799-6 Sugaya, Naka-shi, Ibaraki 311-0105, Japan; sizuko-masa@themis.ocn.ne.jp

**Keywords:** mixed dish, dietary pattern, diet quality, meal, HEI-2015, NRF9.3, Japan

## Abstract

We identified dish-based dietary patterns for breakfast, lunch, and dinner and assessed the diet quality of each pattern. Dietary data were obtained from 392 Japanese adults aged 20–69 years in 2013, using a 4 d dietary record. *K*-means cluster analysis was conducted based on the amount of each dish group, separately for breakfasts (*n* = 1462), lunches (*n* = 1504), and dinners (*n* = 1500). The diet quality of each dietary pattern was assessed using the Healthy Eating Index 2015 (HEI-2015) and Nutrient-Rich Food Index 9.3 (NRF9.3). The extracted dietary patterns were as follows: ‘bread-based’ and ‘rice-based’ for breakfast; ‘bread’, ‘rice-based’, ‘ramen’, ‘udon/soba’, and ‘sushi/rice bowl dishes’ for lunch; and ‘miscellaneous’, ‘meat dish and beer’, and ‘hot pot dishes’ for dinner. For breakfast, the HEI-2015 and NRF9.3 total scores were higher in the ‘rice-based’ pattern than the ‘bread-based’ pattern. For lunch, the HEI-2015 and NRF9.3 total scores were relatively high in the ‘rice-based’ pattern and low in the ‘ramen’ pattern. For dinner, the HEI-2015 total score was the highest in the ‘meat dish and beer’ pattern, and the NRF9.3 total score was higher in the ‘hot pot dishes’ than the ‘miscellaneous’ pattern. These results suggested that breakfast, lunch, and dinner have distinctive dietary patterns with different diet qualities.

## 1. Introduction

Although many studies in nutritional epidemiology have focused on single foods and specific nutrients, this approach may be inappropriate in considering synergistic and interactive effects among foods and nutrients [1,2,3]. Therefore, a growing interest in dietary pattern analysis considers overall diet rather than individual foods or nutrients. Since dietary patterns may be easy for the public to translate into diets, dietary pattern analysis can be useful in developing public health implications [1]. Furthermore, because dietary patterns are characterised on the basis of eating behaviour, they can also facilitate understandings of dietary practice, and provide guidance for nutritional intervention and education [1].

Most work on dietary patterns has analysed overall dietary intake without differentiating eating occasions. However, since people eat foods in specific combinations at mealtimes [4,5,6,7,8], grouping breakfast, lunch, and dinner together may overlook the distinctive dietary patterns of each eating occasion [4]. To date, there have been several studies investigating meal-specific dietary patterns for breakfast [9,10] and all main meals (breakfast, lunch, or dinner) [4,11,12,13]. These studies have indicated associations of specific dietary patterns at breakfast, lunch, or dinner with an overall dietary pattern [11], overall diet quality [9], and risk of hyperglycaemia [12].

Moreover, previous dietary pattern analyses have been conducted at the level of single foods or food groups, whereas people generally eat mixed dishes rather than single foods [14]. Mixed dishes reflect not only the quantities and combinations of individual foods, but also the varieties of elements related to eating behaviours, such as cooking practice, serving, and eating situations. Therefore, identification of dietary patterns at the level of mixed dishes, rather than single foods, would help us better understand human eating behaviours at meals. Furthermore, given that mixed dishes are the state of foods consumed, assessing dish-based dietary patterns at different eating occasions in relation to diet quality may be useful to develop practical dietary recommendations that people can easily understand.

However, to our knowledge, no study has investigated meal-specific dietary patterns based on mixed dishes. Therefore, the aim of the present study was to identify dish-based dietary patterns for breakfast, lunch, and dinner, and to assess the diet quality of each dietary pattern.

## 2. Materials and Methods

### 2.1. Data Source

This cross-sectional study was based on data from a nationwide dietary survey conducted in 20 study areas consisting of 23 prefectures in Japan between February and March 2013. Details of the survey have been described elsewhere [15,16]. In brief, 199 research dietitians working at separate welfare facilities recruited their colleagues and the family members of the colleagues as study participants. The recruitment was conducted such that each study area included approximately four apparently healthy subjects (two men and two women) from each of five 10-year age categories (20–29, 30–39, 40–49, 50–59, and 60–69 years). One individual per household could participate in the survey. None of the participants was a dietitian or a medical professional, had received dietary therapy by a doctor or dietitian, had a history of educational hospitalisation for diabetes mellitus, or was a pregnant or lactating woman. In total, 196 men and 196 women aged 20–69 years provided dietary data for the analysis. Body weight (to the closest 0.1 kg) in light clothes and body height (to the closest 0.1 cm) without shoes was measured using standardised procedures by research dietitians or medical workers. Body mass index (BMI) was calculated as body weight (kg) divided by the square of body height (m^2^).

### 2.2. Dietary Assessment

Dietary data were obtained using a 4-day weighed dietary record (DR), as described previously [16]. Briefly, participants were asked to record all foods and beverages consumed for four non-consecutive days (three working days and one non-working day, excluding days before and after a night shift). Research dietitians explained to the participants how to keep the DR and requested them to weigh foods and beverages with a provided digital scale or measuring spoon and cup. The main recorded items were dish names, food names (including beverages and any ingredients in dishes), and approximate amounts or measured weights of foods consumed. The participants submitted the recording sheets to a research dietitian at each facility shortly after recording. The research dietitians reviewed the recording sheets as soon as possible, and if necessary, asked participants additional information to clarify the name or amount of food on the sheet. The research dietitian at each facility coded each food item recorded in the column ‘food names’ using the Standard Tables of Food Composition in Japan [17] in a uniform procedure. All food codes and weights were then reconfirmed by two other research dietitians at the central office of the study.

### 2.3. Definition of Breakfast, Lunch, and Dinner

The DR sheet consisted of four sections (breakfast, lunch, dinner, and snacks). The identification of eating occasions was based on this classification; thus, a meal recorded in the sections of breakfast, lunch, or dinner on the recording sheet was considered the respective meal. Eating occasions including only water or dietary supplements were excluded in this study. In total, the number of meals was 1474 for breakfast, 1515 for lunch, and 1551 for dinner.

### 2.4. Identification of Dish Groups

To classify dish items consumed at each eating occasion into dish groups, each unique dish item in the column ‘dish items’ was coded using a recently developed dish composition database [18,19]. A detailed description of the database has been reported elsewhere [18,19]. Briefly, the dish composition database was developed based on a 16-day weighed DR completed by 252 Japanese adults. For a total of 371 dish groups, the database consisted of information on dish names, dish codes, portion size, and food groups and nutrient contents.

In this study, a total of 20,057 dishes (5492 for breakfast, 7263 for lunch, and 7302 for dinner) appeared in the DR. We coded each dish item using dish codes in the dish composition database, and consequently, all dishes were classified into 335 dish groups. To remove the complexity, these dish groups were further consolidated into 59 dish groups for the present analysis, based on similarity of dish name, ingredients, cooking methods, and energy and nutrient content (Supplementary Table S1). Refined grain dishes and wholegrain dishes were not separated in the process of grouping dishes, since wholegrain intake is very low in Japan [8,11]. After this process, the number of dish groups included in breakfast, lunch, and dinner was 54, 59, and 59, respectively.

### 2.5. Calculation of Diet Quality Scores

Estimates of energy and nutrient content for individual meals were calculated based on the weight of food items and their nutrient content, using the Standard Tables of Food Composition in Japan [17]. For food items with unavailable data on the added sugar content in that database, added sugar values were calculated based on the same or similar food in the 2011–2012 Food Patterns Equivalents Database [20]. Teaspoon equivalents in the Food Patterns Equivalents Database were converted into grams by multiplying by 4.2 (grams of added sugar per teaspoon).

The diet quality of individual meals was assessed using the Healthy Eating Index 2015 (HEI-2015) [21,22] and Nutrient-Rich Food Index 9.3 (NRF9.3) [23,24]. These indices have been used to assess not only overall diet quality [25], but also individual meals [8,26,27,28,29]. The usefulness of HEI-2015 and NRF9.3 has been demonstrated in the Japanese population [30]. The HEI-2015 is a 100-point scale designed to assess the conformance of a set of foods with the 2015–2020 Dietary Guidelines for Americans [22,31], with a higher score indicating a better quality of diet. The nine adequacy components (maximum score) include total fruits (5), whole fruits (5), total vegetables (5), greens and beans (5), wholegrains (5), dairy (10), total protein (5), seafood and plant protein (5) and fatty acids (the ratio of the sum of polyunsaturated and monounsaturated fatty acids to saturated fatty acids) (10); the four moderation components include refined grains (10), sodium (10), added sugars (10) and saturated fats (10). We calculated the total and component scores of HEI-2015 based on the energy-adjusted values in each meal (i.e., amount/4184 kJ of energy or percent of energy), except for fatty acids, using the 2011–2012 Food Patterns Equivalents Database [20].

The NRF9.3 is a composite measure of nutrient density, which can be applied to individual foods, meals, and menus or to the total diet [23,24]. The NRF9.3 is calculated as the sum of the percentage of reference daily values (RDVs) for nine qualifying nutrients minus the sum of the percentage of RDVs for three disqualifying nutrients. For qualifying nutrients, the RDV percentage of each was truncated at 100, so that a high intake of one nutrient could not compensate for a low intake of another. As such, the maximum possible score is 900, with the higher NRF9.3 total score indicating a better quality of diet. RDVs were determined based on the Dietary Reference Intakes (DRIs) for Japanese, 2020 [32], except for added sugar (Supplementary Table S2). The Japanese DRIs present different values depending on sex and age categories. In this study, we used the RDVs for men aged 30–49 years to evaluate all meals uniformly. We used the Recommended Dietary Allowance for protein, vitamins A and C, calcium, iron, and magnesium, the tentative dietary goal for preventing lifestyle-related diseases for dietary fibre, potassium, saturated fats, and sodium, and the adequate intake for vitamin D. For added sugar, because the intake is low [33] and no recommended amount is shown in Japan, we used the conditional recommendation advocated by the World Health Organisation (i.e., upper limit of 5% of energy) [34]. For added sugar and saturated fats (RDVs are calculated as a percent of energy), the energy intake was determined from Estimated Energy Requirement for the moderate level of physical activity in the DRIs. We calculated the total and component scores of NRF9.3 based on nutrient content for individual meals, which was adjusted for the energy content of each meal by the density method.

### 2.6. Data Analysis

All analyses were conducted using the statistical software package SAS version 9.4 (SAS Institute Inc., Cary, NC, USA). For simplicity and ease of interpretation, dish groups representing less than 3% of the total number of meals at breakfast, lunch, and dinner were removed from the analysis. We calculated the consumption frequency of each dish group by dividing the number of dishes by the total number of meals separately for breakfast, lunch, and dinner. Cluster analysis was performed to divide individual meals into mutually exclusive groups based on the similarity of the amount of dish groups. We used the *k*-means method, which uses Euclidean distances between each meal to estimate clusters empirically from the data set, to avoid imposing a hierarchical structure on the clusters [35]. The input variables were the wet amount of dish groups (grams) in each meal. The variables were not standardised before entering into cluster analysis, because all the variables had the same unit (grams), and standardisation would dilute differences between clusters and reduce correlations between variables [35]. The FASTCLUS procedure in SAS was performed separately for breakfast, lunch, and dinner. As cluster analysis is sensitive to outliers, we removed them using the following two methods. First, we removed meals with a dish group whose amount was 5 standard deviations away from the mean of that dish group [36,37]. Second, we ran cluster analysis with a predefined number of 20 clusters for each meal, and removed meals in clusters with less than 8 meals [36]. Through these procedures, a total of 74 meals were removed, leaving the final samples of 1462, 1504, and 1500 meals for breakfast, lunch, and dinner, respectively.

The number of clusters had to be determined in the *k-*means method prior to analysis. To determine the most appropriate number of clusters, 2 to 8 cluster solutions were examined. The final cluster solution was selected based on a plot of the ratio of between-cluster variance to within-cluster variance vs. the number of clusters [38,39,40], a plot of within-cluster variance vs. the number of clusters [40], cubic clustering criterion [39,41], the sample size (cluster solutions were retained only if each cluster contained 5% of the total sample in each meal type) [42], and the interpretation of each cluster [41,43]. The reproducibility of the clusters was assessed separately for breakfast, lunch, and dinner, by performing the *k*-means cluster analysis repeatedly with the selected number of cluster solutions in random samples of 50% of the participants [43]. We confirmed that similar results were identified to those in the original analysis.

To describe the characteristics of each cluster, we calculated the means and standard errors of the amount of food groups, dish groups, and the total and component scores of HEI-2015 and NRF9.3 for each cluster in breakfast, lunch, and dinner. Food groups were based mainly on the Standard Tables of Food Composition in Japan [17] and the similarity of nutrient composition or culinary use of foods (Supplementary Table S3). Differences in the mean values of the amount of each food group and dish group, as well as the HEI-2015 and NRF9.3 total and component scores across clusters, were compared for each eating occasion using an independent t-test, or ANOVA followed by Tukey’s test when appropriate. The clusters were basically labelled based on the dish groups with a significantly larger mean amount than any other clusters within each eating occasion. Two-sided *p*-values < 0.05 were considered statistically significant.

## 3. Results

Participants characteristics of the study participants have been provided elsewhere [16]. The present analysis included 1587 days of dietary data obtained from 392 Japanese adults with a mean age of 44.5 years (SD 13.4) and a mean BMI of 23.3 kg/m^2^ (SD 3.6). Of the 59 dish groups, the numbers of dish groups consumed in ≥3% of the total number of meals were 24, 37, and 37 for breakfast, lunch, and dinner, respectively.

Table 1 shows the consumption frequency of dish groups with a consumption frequency of 3% or more in all breakfasts, and the weight (g) of dish groups in each dietary pattern. The most frequently consumed dish groups were Japanese and Chinese tea, coffee/tea, rice, plain bread, and miso soup.

The cluster analysis identified two dietary patterns. The predominant pattern (*n* = 983) was characterised by larger amounts of coffee/tea, plain bread, sweet bread/savoury bread, milk/soymilk, Western or Chinese soup, and ice cream/jelly/pudding/cake. Since there were many dish groups with a larger amount of consumption including bread, this pattern was named the ’bread-based’ pattern. Meanwhile, the other pattern (*n* = 479), which was named the ‘rice-based’ pattern, was characterised by larger amounts of Japanese and Chinese tea, rice, miso soup, egg dishes, tsukudani/pickles, natto (fermented soybeans), grilled or stir-fried seafood dishes, rice ball, Japanese-style vegetable side dishes, mushrooms/seaweed/konnyaku dishes, and canned fish/seafood delicacy.

For lunch, the most frequently consumed dish groups were Japanese and Chinese tea, rice, raw or boiled vegetable dishes, miso soup, and fruit/fruit dishes (Table 2). Five clusters were identified. The ‘bread’ pattern (*n* = 781) was characterised by the largest amount of sweet bread/savoury bread and the smallest amount of Japanese and Chinese tea. The ‘rice-based’ pattern (*n* = 393) was characterised by the largest amounts of Japanese and Chinese tea, rice, egg dishes, and grilled or stir-fried meat dishes. Each of the other three clusters had one dish group with the largest amount: namely, the ‘ramen’ (*n* = 99), ‘udon/soba’ (*n* = 125), and ‘sushi/rice bowl dishes’ (*n* = 106) patterns.

For dinner, the most frequently consumed dish groups were Japanese and Chinese tea, rice, miso soup, raw or boiled vegetable dishes, and Japanese-style vegetable side dishes (Table 3). Three dietary patterns were identified. Most meals were categorised into the ‘miscellaneous’ pattern (*n* = 1211), which did not have any specific dish groups with a significantly larger amount than any other clusters. The ‘meat dish and beer’ pattern (*n* = 165) was characterised by the largest amounts of beer, gyoza dumplings/shumai/meatballs, and simmered meat with seasoning, and the smallest amounts of Japanese and Chinese tea and rice. The ‘hot pot dishes’ pattern (*n* = 124) was characterised by the largest amount of hot pot dishes, and the smallest amounts of miso soup, raw or boiled vegetable dishes, Japanese-style vegetable side dishes, grilled or stir-fried seafood dishes, grilled or stir-fried meat dishes, deep-fried meat dishes, and curry and rice/omelette rice.

The total and component scores of HEI-2015 and NRF9.3 for each dietary pattern are summarised in Table 4. For breakfast, the ‘rice-based’ pattern scored higher in the total score of HEI-2015 and its component scores for total vegetable, greens and beans, total protein foods, seafood and plant proteins, fatty acids, added sugars, and saturated fats. Meanwhile, the ‘bread-based’ pattern had higher HEI-2015 component scores for total fruits, whole fruits, dairy, refined grains, and sodium. For NRF9.3, the ‘rice-based’ pattern was higher in the total score and the component scores of protein, dietary fibre, vitamin A, vitamin C, iron, and sodium, while the ‘bread-based’ pattern had higher component scores for added sugars and saturated fats.

For lunch, the HEI-2015 total score was higher in the ‘rice-based’ pattern than the ‘bread’, ‘ramen’, and ‘udon/soba’ patterns, and the lowest in the ‘ramen’ pattern. For the HEI-2015 component scores, the ‘bread’ pattern was the highest in dairy, and the ‘udon/soba’ was the highest in wholegrains. The NRF9.3 total score was higher in the ‘bread’, ‘rice-based’, and ‘sushi/rice bowl dishes’ patterns than the other two patterns. For the NRF 9.3 component scores, the ‘rice-based’ pattern was the highest in vitamin C, and the ‘ramen’ pattern was the highest in sodium. The ‘sushi/rice bowl dishes’ pattern had the lowest score for dietary fibre.

For dinner, the ‘meat dish and beer’ pattern was the highest in the HEI-2015 total score and the component scores for refined grains, sodium, and saturated fats. The ‘miscellaneous’ pattern was the lowest in the component score for refined grains. The ‘hot pot dishes’ pattern had the highest component scores for total vegetables and total protein foods, and the lowest component scores for fatty acids and saturated fats. The NRF9.3 total score was higher in the ‘hot pot dishes’ pattern than the ‘miscellaneous’ pattern. The ‘meat dish and beer’ pattern had the lowest component scores in many components (including protein, dietary fibre, vitamin A, calcium, iron, saturated fats, and sodium). In contrast, the ‘hot pot dishes’ pattern had the highest component scores in many components (including dietary fibre, vitamin C, calcium, iron, potassium, and saturated fats). The weight (g) of food groups for each dietary pattern is summarised in Supplementary Table S4.

## 4. Discussion

In the present study, we identified different numbers of dietary patterns for each eating occasion: two for breakfast, five for lunch, and three for dinner. Each of the dietary patterns differed in amount and type of dish groups as well as diet quality. Although there have been several studies that investigated meal-specific dietary patterns at breakfast level or main-meal level [4,9,10,11,12,13], they have analysed meals at the level of single foods or food groups. Moreover, overall diet quality has not been assessed for each dietary pattern. To our knowledge, this is the first study to investigate dish-based dietary patterns and the diet quality of each pattern for breakfast, lunch, and dinner.

Previous studies have shown that some food groups were commonly consumed at all three main meals in Japan, such as rice, vegetables, tea, and coffee, whereas the consumption frequency and amount of many other food groups differed among eating occasions [5,8,11]. Similarly, in this study, there are some common dish groups in the top five most frequently consumed items on each eating occasion (i.e., Japanese and Chinese tea, rice, and miso soup), while the consumption frequency of many dish groups differed among eating occasions. For instance, milk/soymilk, cocoa/milk beverage, and vegetable and fruit juice were frequently consumed at breakfasts, fried rice/pilaf/doria was frequently consumed at lunch, and raw fish dishes and alcoholic beverages (beer, Japanese sake/shochu, and fruit liquor/wine) were frequently consumed at dinner. Consequently, separate dietary patterns with different levels of diet quality scores were derived for each eating occasion. A previous study conducted in Germany has also shown that the consumption frequency of food groups varied across eating occasions [4]. These results support the appropriateness and importance of dietary analysis focusing on each eating occasion.

The number of dietary patterns identified in this study was the smallest in breakfast. This is consistent with a previous study conducted in Brazil [13], whereas other studies have derived equal numbers of dietary patterns for each eating occasion (two patterns in China and four patterns in Japan) [11,12]. Our results showed that dietary patterns at breakfast and lunch were mainly characterised by staple foods (i.e., bread, rice, or noodles). In addition, several dietary patterns were similar between breakfast and lunch, namely the ‘bread-based’ pattern for breakfast and the ‘bread’ pattern for lunch, and the ‘rice-based’ patterns for breakfast and lunch. The other dietary patterns for lunch were characterised by ramen, udon/soba, and sushi/rice bowl dishes, which were rarely eaten at breakfast (consumption frequency < 1.2%). A previous study on food group intake has also identified dietary patterns characterised by rice or bread for breakfast, and those characterised by bread or noodles for lunch among Japanese adults [11]. These results reveal distinctive dietary patterns in relation to eating occasions among Japanese people. Meanwhile, for dinner, although the ‘meat dish and beer’ pattern was characterised by a relatively small content of rice, none of the dietary patterns were characterised by a large amount of staple foods. Rather, dietary patterns at dinner were particularly characterised by other dish groups, such as alcoholic beverages and hot pot dishes.

For breakfast, the number of meals of the ‘bread-based’ pattern was twice that of the ‘rice-based’ pattern. Previous studies conducted in Mexico and the United States have also shown several dietary patterns for breakfast characterised by bread, while there has been no dietary pattern characterised by rice [9,10]. The ‘rice-based’ pattern in breakfast had similar characteristics to the ‘Japanese’ pattern identified in a previous study, which was characterised by larger intakes of mushrooms, seaweeds, potatoes, vegetables, pickles, pulses, seasonings, fruits, and fish and shellfish [44]. Both the HEI-2015 and NRF9.3 showed that overall diet quality was higher in the ‘rice-based’ pattern than the ‘bread-based’ pattern at breakfast. The ‘rice-based’ pattern had higher HEI-2015 component scores for total vegetable, greens and beans, total protein foods, and seafood and plant proteins. This may be due to large amounts of Japanese-style vegetable side dishes: mushrooms/seaweed/konnyaku dishes, natto (fermented soybeans), egg dishes, grilled or stir-fried seafood dishes, and canned fish/seafood delicacy. However, both diet quality measures showed that the ‘rice-based’ pattern in breakfast contained higher sodium than the ‘bread-based’ pattern. A previous study analysing sodium sources in the same population has revealed that the top contributor to total sodium intake was seasonings, followed by fish and shellfish [16]. These foods are the main ingredients of the dish groups characterising the ‘rice-based’ pattern, such as miso soup, tsukudani (fish, meat, or seaweeds simmered in soy sauce) and pickles, and canned fish/seafood delicacy (including salted fish and shellfish). Traditionally, rice is eaten together with such salty foods in Japan. Therefore, reducing these salty dishes would be a key to improving diet quality in the ‘rice-based’ pattern. On the other hand, while the ‘bread-based’ pattern had less sodium content, this pattern contained higher amounts of added sugars and saturated fats than the ‘rice’ pattern. This may be because this pattern was characterised by dish groups that are the major sources of sugars and fats, i.e., coffee/tea (including those with sugar and milk), plain bread (including those with butter, margarine, or jam), sweet bread/savoury bread, milk/soymilk, and ice cream/jelly/pudding/cake.

As with the breakfast dietary pattern, the predominant pattern in lunch was the ‘bread’ pattern, followed by the ‘rice-based’ pattern. In comparison, the numbers of meals of the ‘udon/soba’, ‘sushi/ricebowl dishes’, and ‘ramen’ patterns were small. The ‘rice-based’ pattern had a relatively high overall diet quality assessed by the HEI-2015 and NRF9.3. The ‘rice-based’ pattern in lunch had similar sodium content to that in breakfast. However, sodium content was much higher in the ‘ramen’ and ‘udon/soba’ patterns. This may be attributable to the noodle itself and noodle soup [16]. The ‘ramen’ pattern had the lowest overall diet quality assessed by HEI-2015 among lunch dietary patterns. Compared to the ‘rice-based’ pattern, the ‘ramen’ pattern had lower amounts of dish groups including egg dishes, potato dishes, grilled or stir-fried meat dishes, simmered vegetable dishes, and grilled or stir-fried seafood dishes. As such, it seems that ramen is not eaten in combination with main or side dishes consisting of a variety of foods, possibly resulting in the low HEI-2015 component scores for total vegetables, greens and beans, total protein foods, and seafood and plant proteins. In addition, the ‘ramen’ pattern also had lower overall dietary quality assessed by NRF9.3 among the lunch patterns, and the same was true with the ‘udon/soba’ pattern, another noodle-based dietary pattern. Nevertheless, the ‘udon/soba’ pattern had the highest HEI component scores of wholegrain, mainly because of soba (buckwheat noodle).

For dinner, most meals (81%) were categorised into the ‘miscellaneous’ dietary pattern. This pattern contained a larger amount of rice and had the lowest HEI component score for refined grains. On the other hand, the ‘meat dish and beer’ pattern, which had the smallest amount of rice, had the highest component score for refined grain, and also had the highest HEI total score. The ‘meat dish and beer’ pattern was also characterised by the largest amounts of meat dishes, including gyoza dumplings/shumai/meatballs and simmered meat with seasoning. Nevertheless, it showed the highest quality (namely, lowest content) for saturated fats in both HEI-2015 and NRF9.3. Rather, the ‘hot pot dishes’ pattern had a lower score for saturated fats in both diet quality measures. This may be because hot pot dishes (including shabu-shabu and sukiyaki) consist of a lot of meat (thinly sliced pork or beef). Moreover, since hot pot dishes include various kinds of vegetables, the ‘hot pot dishes’ pattern had the highest HEI-2015 component scores for total vegetables and total protein foods, despite the smallest amounts of stir-fried meat or seafood dishes as well as vegetable dishes. The ‘hot pot dishes’ pattern also had relatively high diet quality, as assessed by NRF 9.3.

A strength of this study is its examination of meal-specific dietary patterns at the level of mixed dishes, using detailed dietary information obtained from a 4 d DR. Importantly, this approach enabled us to reveal dish-based dietary patterns that could not be identified when looking at individual food intake. Since mixed dishes are the state of foods closer to that in a real eating situation, dish-based dietary pattern analysis focusing on eating occasions would provide an important basis for elucidating people’s dietary behaviours and developing practical dietary advice. For instance, it would be important to reduce salty dishes for rice-based meals, and sugary or fatty dishes for bread-based meals, for better diet quality at breakfast. In addition, when eating ramen for lunch, increasing the variety of foods by adding side dishes would be important. Such practical advice can be useful to develop public health policy to facilitate healthier diet choices. Moreover, we used cluster analysis to group meals based on the similarity in the amounts of dish groups, which enabled us to clarify dish groups consumed together at meals. However, given that most dinners were classified into the ‘miscellaneous’ dietary pattern, dietary patterns may not have been clearly separated for dinner, possibly due to the small sample size. Thus, increasing sample size or using other approaches, such as principal component analysis, should be considered in future research.

Several limitations of this study should be mentioned. First, the DR was obtained over four days per person, which may have reduced the variation in types of dish groups and dietary patterns. Second, since the DR was obtained only in winter, seasonal variation in mixed dishes was not reflected in dietary data. For example, hot pot dishes, which characterised one of the dietary patterns for dinner, are not frequently eaten in other seasons in general. Given the seasonal variation in dietary intake [45], dish-based dietary patterns should be investigated using dietary data over four seasons in future analysis. Third, self-reported dietary information is subject to both random and systematic errors [46,47], which may have had an influence on deriving dietary patterns [3]. Although we used energy-adjusted values for diet quality measures, the results should be interpreted with caution. Fourth, since the NRF9.3 was calculated based on the RDVs for men aged 30–49 years uniformly (except for added sugar), the nutritional quality of individual meals may have not been accurately evaluated. However, this may not have greatly influenced the study results, given that NRF9.3 reflects the nutrient density of the diet. Moreover, NRF9.3 provided generally similar results to HEI-2015, which was not calculated using the RDVs. Fifth, cluster analysis is hindered by subjective analytical decisions [3], including the number (and grouping) of dish groups, the definition of eating occasions, the form of the input variables, the number of cluster solutions, as well as clustering methods. In addition, although we assessed the reproducibility of the clusters in a random half of the samples, the reproducibility of cluster solutions does not guarantee validity [48]. Sixth, the study participants were not randomly selected, but were healthy volunteers, likely more health-conscious than the general population. Finally, although beyond the scope of the study, we did not examine the differences in dietary patterns related to individual characteristics such as sex, age, and obesity status. Moreover, we did not assess the association between the dish-based dietary patterns of individual meals and the overall dietary quality at the individual level, which was beyond the scope of this study. Thus, it would be of interest to investigate the association of dish-based dietary patterns of individual meals with participant characteristics, or overall diet quality, in future research.

## 5. Conclusions

In conclusion, using a 4 d DR obtained from Japanese adults, we investigated dish-based dietary patterns for breakfast, lunch, and dinner, and assessed their diet quality. The results showed distinctive dietary patterns: two patterns for breakfast, five for lunch, and three for dinner, which may serve to help us understand the meal-specific dietary choices of Japanese people. Moreover, this study provided insight into the health aspects of dietary patterns at each eating occasion. The diet scoring of meal- and dish-based dietary patterns would help to provide specific dietary advice in the meal context so that people can modify their dietary behaviour. Future studies with increased sample sizes and different statistical approaches are required to obtain further insight into dish-based dietary patterns.

## Figures and Tables

**Table 1:** Consumption frequency of dish groups in all breakfasts ^1^ and weight (g) of dish groups in each dietary pattern ^2^.

Dish Group			All Breakfasts (*n* = 1474)	Breakfasts Included in Cluster Analysis ^3^ (*n* = 1462)				
			Frequency	Weight (g)				*p * ^4^
				Bread-Based (*n* = 983)		Rice-Based (*n* = 479)		
ID	Name	*n*	%	Mean	SE	Mean	SE	
48	Japanese and Chinese tea	650	44.1	22.0	1.6	258.8	6.0	<0.0001
47	Coffee/tea	602	40.8	118.5	4.0	22.7	2.9	<0.0001
1	Rice	474	32.2	27.8	2.1	99.1	4.3	<0.0001
8	Plain bread	424	28.8	31.2	1.4	10.5	1.4	<0.0001
55	Miso soup	365	24.8	25.7	2.3	113.6	5.9	<0.0001
27	Fruit/fruit dishes	305	20.7	20.4	1.5	17.3	1.8	0.22
40	Egg dishes	260	17.6	10.2	1.0	18.2	1.7	<0.0001
42	Yogurt/cheese	242	16.4	15.7	1.3	17.6	2.1	0.40
9	Sweet bread/savoury bread	231	15.7	21.2	1.6	10.2	1.7	<0.0001
41	Milk/soymilk	229	15.5	32.9	2.5	20.8	2.7	0.003
22	Raw or boiled vegetable dishes	189	12.8	10.3	1.2	10.3	1.3	0.99
49	Cocoa/milk beverage	146	9.9	17.5	1.9	14.2	2.2	0.29
58	Tsukudani/pickles	144	9.8	1.2	0.2	4.9	0.6	<0.0001
17	Natto (fermented soybeans)	139	9.4	2.8	0.4	8.4	0.9	<0.0001
50	Vegetable and fruit juice	126	8.5	16.9	1.8	14.2	2.6	0.40
39	Processed meat dishes	96	6.5	2.6	0.4	3.4	0.7	0.23
30	Grilled or stir-fried seafood dishes	74	5.0	2.4	0.4	4.6	0.8	0.01
6	Rice ball	72	4.9	6.6	1.1	13.5	2.6	0.005
21	Japanese-style vegetable side dishes	70	4.7	1.4	0.3	4.9	1.0	<0.0001
56	Western or Chinese soup	68	4.6	11.4	1.6	5.2	1.5	0.02
26	Mushrooms/seaweed/konnyaku dishes	52	3.5	0.1	0.1	1.6	0.5	<0.0001
44	Ice cream/jelly/pudding/cake	48	3.3	3.1	0.5	1.2	0.5	0.03
16	Potato dishes	46	3.1	2.4	0.6	2.9	0.8	0.60
33	Canned fish/seafood delicacy	44	3.0	0.3	0.1	0.8	0.2	0.009

SE, standard error. ^1^ The number of dishes per total number of breakfasts. In total, 5492 dishes (54 dish groups) were consumed at breakfast. Only 24 dish groups with a consumption frequency of 3% or more are shown. ^2^ Each cluster represents a group of meals with a similar dish-based dietary pattern. ^3^ Twelve meals were removed from cluster analysis; three meals with a dish group whose amount was five standard deviations away from the mean of that dish group, and nine meals that fell into clusters of less than eight meals when cluster analysis was conducted with a predefined number of 20 clusters. ^4^ Differences between two dietary patterns were tested with an independent t-test.

**Table 2:** Consumption frequency of dish groups in all lunches ^1^ and weight (g) of dish groups in each dietary pattern ^2^.

Dish Group		All Lunches (*n* = 1515)		Lunches Included in Cluster Analysis ^3^ (*n* = 1504)										
		Frequency		Weight (g)										*p*
				Bread (*n* = 781)		Rice-Based (*n* = 393)		Ramen (*n* = 99)		Udon/Soba (*n* = 125)		Sushi/Rice Bowl Dishes (*n* = 106)		
ID	Name	*n*	%	Mean	SE	Mean	SE	Mean	SE	Mean	SE	Mean	SE	
48	Japanese and Chinese tea	968	63.9	67.3 ^a^	2.8	320.2 ^b^	6.9	153.0 ^c^	15.2	142.5 ^c^	12.3	159.3 ^c^	14.3	<0.0001
1	Rice	799	52.7	105.3 ^a^	3.6	148.9 ^b^	5.0	19.0 ^c^	5.8	24.9 ^c^	5.8	0.0 ^c^	0.0	<0.0001
22	Raw or boiled vegetable dishes	437	28.8	22.5 ^a^	1.7	25.9 ^a^	2.6	5.7 ^b^	3.3	9.7 ^b^	2.5	16.3 ^ab^	3.8	<0.0001
55	Miso soup	375	24.8	53.8 ^a^	3.2	51.9 ^a^	4.8	0.0 ^b^	0.0	4.0 ^b^	2.4	66.2 ^a^	8.9	<0.0001
27	Fruit/fruit dishes	326	21.5	16.2	1.2	14.1	1.7	8.0	2.5	10.9	2.5	16.7	3.8	0.12
21	Japanese-style vegetable side dishes	322	21.3	14.7 ^a^	1.1	15.0 ^a^	1.5	4.0 ^b^	1.6	9.2 ^ab^	2.4	16.7 ^a^	4.0	0.005
58	Tsukudani/pickles	262	17.3	2.8 ^ab^	0.3	4.1 ^a^	0.6	2.2 ^ab^	0.8	0.7 ^b^	0.4	3.1 ^ab^	0.8	0.006
47	Coffee/tea	251	16.6	49.2 ^a^	3.6	14.1 ^b^	2.8	25.5 ^ab^	7.4	23.4 ^b^	5.6	17.5 ^b^	6.2	<0.0001
40	Egg dishes	211	13.9	8.4 ^a^	0.9	14.8 ^b^	1.5	0.5 ^c^	0.5	2.1 ^ac^	1.3	4.9 ^ac^	3.6	<0.0001
16	Potato dishes	197	13.0	12.9 ^ab^	1.4	16.6 ^a^	2.8	0.9 ^c^	0.7	3.0 ^bc^	1.4	5.3 ^ac^	2.3	0.0002
37	Grilled or stir-fried meat dishes	187	12.3	15.6 ^a^	1.7	24.0 ^b^	3.1	0.8 ^c^	0.8	0.0 ^c^	0.0	1.6 ^c^	1.4	<0.0001
38	Deep-fried meat dishes	171	11.3	11.4 ^ab^	1.3	17.1 ^a^	2.5	0.8 ^bc^	0.6	2.6 ^bc^	1.5	0.5 ^c^	0.5	<0.0001
23	Simmered vegetable dishes	171	11.3	11.4 ^ab^	1.4	15.4 ^a^	2.2	0.0 ^c^	0.0	2.8 ^bc^	1.5	4.6 ^ac^	2.1	0.0001
30	Grilled or stir-fried seafood dishes	167	11.0	9.7 ^ab^	1.1	12.8 ^a^	1.8	0.0 ^c^	0.0	1.4 ^c^	0.9	3.2 ^bc^	1.6	<0.0001
10	Udon/soba	166	11.0	3.7 ^a^	1.0	9.5 ^a^	2.3	0.0 ^a^	0.0	466.8 ^b^	14.1	11.0 ^a^	5.8	<0.0001
56	Western or Chinese soup	144	9.5	24.4 ^a^	2.7	15.0 ^ab^	2.8	2.6 ^b^	2.4	4.2 ^b^	2.5	13.5 ^ab^	5.1	0.0003
5	Sushi/rice bowl dishes	140	9.2	2.4 ^a^	0.7	4.8 ^a^	1.6	1.1 ^a^	1.1	11.2 ^a^	4.2	405.2 ^b^	15.8	<0.0001
9	Sweet bread/savoury bread	122	8.1	17.3 ^a^	1.9	6.8 ^b^	1.7	3.6 ^b^	2.2	4.6 ^b^	2.3	1.4 ^b^	1.2	<0.0001
12	Ramen	114	7.5	2.2 ^a^	0.8	2.8 ^a^	1.4	505.2 ^b^	17.6	0.0 ^a^	0.0	6.1 ^a^	4.3	<0.0001
24	Grilled or stir-fried vegetable dishes	113	7.5	10.1 ^a^	1.5	4.9 ^ab^	1.1	2.8 ^ab^	1.7	2.2 ^ab^	1.6	0.6 ^b^	0.6	0.002
6	Rice ball	105	6.9	12.8 ^ab^	2.0	14.2 ^ab^	2.9	18.2 ^ab^	5.5	22.8 ^a^	5.8	0.0 ^b^	0.0	0.03
44	Ice cream/jelly/pudding/cake	101	6.7	5.3	0.9	4.3	1.0	10.7	4.0	5.7	2.1	7.1	2.1	0.19
32	Fish jelly product dishes	97	6.4	4.7	0.9	5.8	1.6	2.8	1.6	1.7	0.9	2.2	1.8	0.42
18	Tofu dishes	94	6.2	9.1	1.4	7.5	1.8	3.1	1.6	1.0	1.0	7.8	3.1	0.13
39	Processed meat dishes	89	5.9	2.3	0.4	4.4	0.8	1.7	1.0	0.9	0.9	1.2	1.2	0.02
31	Deep-fried fish dishes	86	5.7	5.5	0.9	4.7	1.1	1.5	1.1	2.3	1.5	0.2	0.2	0.06
42	Yogurt/cheese	78	5.1	4.6	0.8	2.2	0.7	3.5	1.7	5.1	1.8	5.0	2.0	0.28
35	Gyoza dumplings/shumai/meatballs	72	4.8	2.0	0.4	3.9	1.0	3.7	1.7	0.0	0.0	0.6	0.6	0.02
3	Curry and rice/omelette rice	64	4.2	26.8 ^a^	4.0	13.9 ^ab^	3.8	2.5 ^ab^	2.5	0.0 ^b^	0.0	0.0 ^b^	0.0	0.0004
26	Mushrooms/seaweed/konnyaku dishes	64	4.2	2.3	0.4	2.5	0.7	0.8	0.8	0.6	0.6	1.5	1.5	0.43
36	Simmered meat with seasoning	60	4.0	9.4	1.7	6.0	1.8	0.0	0.0	0.0	0.0	0.0	0.0	0.007
43	Japanese confectionery	57	3.8	2.2	0.5	1.5	0.7	3.3	1.7	0.7	0.6	1.7	0.8	0.56
29	Seafood dishes (steamed/boiled/ simmered)	56	3.7	4.7 ^ab^	0.9	8.9 ^a^	2.2	0.0 ^ab^	0.0	0.0 ^b^	0.0	2.7 ^ab^	2.4	0.009
8	Plain bread	53	3.5	5.5 ^a^	0.9	1.0 ^b^	0.4	0.7 ^ab^	0.5	0.2 ^b^	0.2	0.0 ^b^	0.0	<0.0001
11	Pasta	50	3.3	12.7	2.5	10.7	3.0	0.0	0.0	0.0	0.0	4.4	4.4	0.07
4	Fried rice/pilaf/doria	48	3.2	10.8	2.1	5.3	1.8	4.2	3.3	2.8	2.8	3.5	2.9	0.14
57	Hot pot dishes	45	3.0	11.0	2.3	10.8	2.9	0.0	0.0	1.9	1.9	5.2	3.9	0.19

SE, standard error. ^1^ The number of dishes per total number of lunches. In total, 7263 dishes (59 dish groups) were consumed at lunch. Only 37 dish groups with a consumption frequency of 3% or more are shown. ^2^ Each cluster represents a group of meals with a similar dish-based dietary pattern. Mean values between clusters without common letter differ, *p* < 0.05 tested with ANOVA and Tukey post-hoc tests. ^3^ Eleven meals were removed from the cluster analysis; 10 meals with a dish group whose amount was five standard deviations away from the mean of that dish group, and one meal that fell into clusters of less than eight meals when cluster analysis was conducted with a predefined number of 20 clusters.

**Table 3:** Consumption frequency of dish groups in all dinners ^1^ and weight (g) of dish groups in each dietary pattern ^2^.

Dish Group		All Dinners (*n* = 1551)		Dinners Included in Cluster Analysis ^3^ (*n* = 1500)						
		Frequency		Weight (g)						*p*
				Miscellaneous (*n* = 1211)		Meat Dish and Beer (*n* = 165)		Hot Pot Dishes (*n* = 124)		
ID	Name	*n*	%	Mean	SE	Mean	SE	Mean	SE	
48	Japanese and Chinese tea	909	58.6	166.3 ^a^	4.8	72.1 ^b^	10.2	162.7 ^a^	16.1	<0.0001
1	Rice	874	56.4	106.2 ^a^	3.1	62.3 ^b^	7.1	102.0 ^a^	10.2	<0.0001
55	Miso soup	464	29.9	79.7 ^a^	3.7	73.4 ^a^	10.0	4.0 ^b^	2.3	<0.0001
22	Raw or boiled vegetable dishes	459	29.6	36.1 ^a^	2.0	46.7 ^a^	6.2	17.3 ^b^	4.8	0.002
21	Japanese-style vegetable side dishes	282	18.2	15.5 ^a^	1.1	18.2 ^a^	3.8	6.8 ^b^	2.1	0.03
30	Grilled or stir-fried seafood dishes	265	17.1	17.9 ^a^	1.4	18.6 ^a^	3.3	5.6 ^b^	2.1	0.01
37	Grilled or stir-fried meat dishes	258	16.6	35.6 ^a^	2.8	42.3 ^a^	7.3	3.3 ^b^	2.2	0.0006
58	Tsukudani/pickles	220	14.2	4.1	0.4	5.1	1.2	5.1	1.6	0.53
53	Beer	206	13.3	2.3 ^a^	0.7	604.8 ^b^	24.8	35.6 ^c^	11.0	<0.0001
16	Potato dishes	179	11.5	17.3 ^a^	1.7	18.7 ^ab^	5.0	4.5 ^b^	2.8	0.053
57	Hot pot dishes	173	11.2	5.9 ^a^	1.0	18.2 ^a^	7.2	640.5 ^b^	21.6	<0.0001
27	Fruit/fruit dishes	169	10.9	10.3	1.0	14.7	3.6	8.0	2.9	0.24
38	Deep-fried meat dishes	152	9.8	14.9 ^a^	1.5	17.9 ^a^	4.8	1.6 ^b^	1.1	0.02
5	Sushi/rice bowl dishes	147	9.5	40.6 ^a^	3.7	31.8 ^ab^	8.5	4.1 ^b^	2.4	0.006
18	Tofu dishes	147	9.5	20.5 ^a^	2.0	7.9 ^ab^	2.8	3.4 ^b^	2.3	0.003
24	Grilled or stir-fried vegetable dishes	139	9.0	13.3 ^a^	1.4	13.0 ^ab^	3.3	1.3 ^b^	1.3	0.03
23	Simmered vegetable dishes	118	7.6	10.5	1.3	13.9	4.3	12.9	7.6	0.66
52	Japanese sake/shochu	114	7.4	17.7 ^a^	3.1	73.5 ^b^	11.9	72.8 ^b^	19.9	<0.0001
56	Western or Chinese soup	113	7.3	19.0 ^a^	2.1	8.5 ^ab^	3.8	1.4 ^b^	1.4	0.005
28	Raw fish dishes	109	7.0	6.5	0.9	9.9	2.6	7.4	3.1	0.43
10	Udon/soba	98	6.3	29.3 ^a^	3.4	28.5 ^ab^	8.7	0.0 ^b^	0.0	0.02
40	Egg dishes	97	6.3	7.5	1.0	10.2	3.1	2.0	1.5	0.13
17	Natto (fermented soybeans)	96	6.2	3.4	0.4	1.4	0.9	2.8	0.9	0.19
47	Coffee/tea	92	5.9	15.6	1.9	4.6	2.3	9.9	3.7	0.08
26	Mushrooms/seaweed/konnyaku dishes	86	5.5	4.7	0.7	5.8	2.2	0.8	0.5	0.15
35	Gyoza dumplings/shumai/meatballs	83	5.4	7.2 ^a^	1.1	15.3 ^b^	4.4	3.0 ^a^	2.0	0.02
29	Seafood dishes (steamed/boiled/ simmered)	82	5.3	10.1	1.4	11.8	3.9	3.4	2.2	0.29
36	Simmered meat with seasoning	79	5.1	14.1 ^a^	2.1	33.4 ^b^	8.8	0.0 ^a^	0.0	0.0006
3	Curry and rice/omelette rice	78	5.0	27.6 ^a^	3.6	37.4 ^a^	10.7	0.0 ^b^	0.0	0.03
54	Fruit liquor/wine	76	4.9	14.6 ^a^	2.4	37.4 ^b^	11.6	29.8 ^ab^	11.9	0.009
31	Deep-fried fish dishes	70	4.5	4.2	0.7	5.7	2.3	2.0	1.0	0.41
44	Ice cream/jelly/pudding/cake	64	4.1	4.0	0.6	3.5	1.4	2.4	1.3	0.73
12	Ramen	57	3.7	22.5	3.4	6.6	4.0	3.8	3.8	0.052
32	Fish jelly product dishes	53	3.4	3.5	0.7	1.7	1.1	3.8	2.3	0.62
33	Canned fish/seafood delicacy	51	3.3	0.8 ^a^	0.2	2.6 ^b^	1.0	0.9 ^ab^	0.4	0.02
43	Japanese confectionery	50	3.2	2.0	0.4	2.3	1.0	0.8	0.8	0.54
11	Pasta	49	3.2	12.0	2.2	10.2	4.6	3.9	3.9	0.47

SE, standard error. ^1^ The number of dishes per total number of dinners. In total, 7302 dishes (59 dish groups) were consumed at dinner. Only 37 dish groups with a consumption frequency of 3% or more are shown. ^2^ Each cluster represents a group of meals with a similar dish-based dietary pattern. Mean values between clusters without a common letter differ, *p* < 0.05 tested with ANOVA and Tukey post-hoc tests. ^3^ Fifty-one meals were removed from cluster analysis; 39 meals with a dish group whose amount was five standard deviations away from the mean of that dish group, and 12 meals that fell into clusters of less than eight meals when cluster analysis was conducted with a predefined number of 20 clusters.

**Table 4:** Comparison of diet quality scores among dish-based dietary patterns for each meal ^1^.

	Breakfast					Lunch											Dinner						
	Bread- Based		Rice- Based		*p*	Bread		Rice- Based		Ramen		Udon/soba		Sushi/Rice Bowl Dishes		*p*	Miscellaneous		Meat Dish and Beer		Hot Pot Dishes		*p*
	(*n* = 983)		(*n* = 479)			(*n* = 781)		(*n* = 393)		(*n* = 99)		(*n* = 125)		(*n* = 106)			(*n* = 1211)		(*n* = 165)		(*n* = 124)		
	Mean	SE	Mean	SE		Mean	SE	Mean	SE	Mean	SE	Mean	SE	Mean	SE		Mean	SE	Mean	SE	Mean	SE	
HEI-2015 (100) ^2^	39.1	0.4	44.3	0.5	<0.0001	44.6 ^a^	0.4	46.5 ^b^	0.5	36.3 ^c^	1.2	43.0 ^a^	1.3	44.3 ^ab^	0.9	<0.0001	46.5 ^a^	0.3	52.6 ^b^	0.7	47.6 ^a^	0.8	<0.0001
Total fruits (5)	1.4	0.1	1.1	0.1	0.004	1.0	0.1	0.7	0.1	0.6	0.1	0.7	0.1	0.8	0.1	0.009	0.5	0.0	0.6	0.1	0.4	0.1	0.34
Whole fruits (5)	1.5	0.1	1.2	0.1	0.02	1.2 ^a^	0.1	1.0 ^ab^	0.1	0.6 ^b^	0.2	0.8 ^ab^	0.2	1.0 ^ab^	0.2	0.005	0.6	0.0	0.6	0.1	0.4	0.1	0.21
Total vegetables (5)	1.8	0.1	3.1	0.1	<0.0001	3.7 ^a^	0.1	3.8 ^a^	0.1	3.0 ^b^	0.2	3.2 ^b^	0.2	3.5 ^ab^	0.2	<0.0001	4.1 ^a^	0.0	4.0 ^a^	0.1	4.8 ^b^	0.1	<0.0001
Greens and beans (5)	0.7	0.1	1.1	0.1	<0.0001	1.7 ^ab^	0.1	2.0 ^b^	0.1	0.8 ^c^	0.2	1.2 ^ac^	0.2	1.7 ^abc^	0.2	<0.0001	1.8	0.1	1.7	0.2	1.7	0.2	0.68
Wholegrains (10)	0.2	0.0	0.1	0.0	0.18	0.2 ^a^	0.0	0.4 ^a^	0.1	0.3 ^a^	0.2	2.0 ^b^	0.4	0.1 ^a^	0.1	<0.0001	0.2	0.0	0.1	0.1	0.1	0.1	0.27
Dairy (10)	4.0	0.1	2.4	0.2	<0.0001	1.1 ^a^	0.1	0.7 ^b^	0.1	0.4 ^b^	0.2	0.5 ^b^	0.1	0.5 ^b^	0.1	<0.0001	0.7	0.1	0.4	0.1	0.5	0.1	0.14
Total protein foods (5)	2.0	0.1	3.4	0.1	<0.0001	3.6 ^a^	0.1	3.9 ^b^	0.1	2.9 ^c^	0.2	2.8 ^c^	0.2	4.4 ^b^	0.1	<0.0001	4.3 ^a^	0.0	4.2 ^a^	0.1	4.8 ^b^	0.1	0.0005
Seafood and plant proteins (5)	1.3	0.1	2.8	0.1	<0.0001	2.7 ^ab^	0.1	2.9 ^ab^	0.1	1.8 ^c^	0.2	2.5 ^ac^	0.2	3.2 ^ab^	0.2	<0.0001	3.3 ^a^	0.1	3.5 ^ab^	0.2	4.0 ^b^	0.2	0.004
Fatty acids (10) ^3^	3.3	0.1	6.4	0.2	<0.0001	6.8 ^a^	0.1	7.4 ^ab^	0.2	6.8 ^ab^	0.4	7.9 ^b^	0.3	6.9 ^ab^	0.3	0.01	7.2 ^a^	0.1	7.3 ^a^	0.3	6.2 ^b^	0.3	0.007
Refined grains (10)	2.4	0.1	1.2	0.1	<0.0001	1.5 ^a^	0.1	1.3 ^a^	0.1	0.3 ^b^	0.1	1.8 ^a^	0.3	1.0 ^ab^	0.2	0.0002	3.0 ^a^	0.1	7.0 ^b^	0.3	5.1 ^c^	0.4	<0.0001
Sodium (10)	6.0	0.1	3.8	0.2	<0.0001	3.5 ^a^	0.1	4.1 ^a^	0.2	0.8 ^b^	0.2	1.3 ^b^	0.3	3.0 ^a^	0.4	<0.0001	2.9 ^a^	0.1	4.4 ^b^	0.3	2.7 ^a^	0.4	<0.0001
Added sugars (10)	7.8	0.1	9.1	0.1	<0.0001	9.0 ^a^	0.1	9.6 ^b^	0.1	9.5 ^ab^	0.2	9.5 ^ab^	0.1	9.2 ^ab^	0.2	<0.0001	9.5 ^a^	0.0	9.9 ^b^	0.0	9.6 ^ab^	0.1	0.01
Saturated fats (10)	6.2	0.1	8.3	0.1	<0.0001	8.6	0.1	8.8	0.1	8.5	0.3	8.8	0.2	9.0	0.2	0.42	8.3 ^a^	0.1	9.0 ^b^	0.2	7.5 ^c^	0.3	0.0002
																							
NRF9.3 (900) 4	421	10	538	8	<0.0001	493 ^a^	11	563 ^b^	9	348 ^c^	24	416 ^c^	20	513 ^ab^	17	<0.0001	536 ^a^	6	559 ^ab^	11	582 ^b^	14	0.02
Protein (100)	95.2	0.4	97.5	0.4	0.0008	96.3 ^a^	0.4	98.1 ^b^	0.4	98.4 ^ab^	0.5	96.7 ^ab^	0.6	98.3 ^ab^	0.6	0.02	98.4 ^a^	0.2	95.0 ^b^	1.1	99.3 ^a^	0.4	<0.0001
Dietary fibre (100)	67.1	1.0	76.5	1.2	<0.0001	74.1 ^a^	1.0	71.5 ^a^	1.4	86.6 ^b^	1.8	83.3 ^b^	1.7	62.8 ^c^	2.8	<0.0001	76.7 ^a^	0.8	62.9 ^b^	2.2	89.0 ^c^	1.7	<0.0001
Vitamin A (100)	48.7	1.1	54.2	1.6	0.005	60.5 ^a^	1.2	60.7 ^a^	1.6	34.5 ^b^	3.3	45.1 ^b^	3.2	59.1 ^a^	3.2	<0.0001	59.6 ^a^	1.0	48.1 ^b^	2.5	63.2 ^a^	3.2	<0.0001
Vitamin C (100)	52.1	1.4	75.6	1.6	<0.0001	76.4 ^a^	1.2	86.0 ^b^	1.4	62.3 ^c^	3.8	62.0 ^c^	3.4	72.5 ^ac^	3.1	<0.0001	81.1 ^a^	0.9	75.8 ^a^	2.3	91.6 ^b^	1.8	<0.0001
Vitamin D (100)	44.2	1.2	44.0	1.7	0.91	42.1 ^a^	1.3	47.7 ^a^	1.9	21.5 ^b^	2.3	27.2 ^b^	2.7	49.9 ^a^	3.3	<0.0001	48.7	1.1	49.7	3.1	48.9	3.0	0.96
Calcium (100)	76.9	0.9	74.8	1.3	0.19	60.7 ^ab^	1.0	58.9 ^ab^	1.4	65.5 ^ab^	2.5	65.9 ^a^	2.6	55.2 ^b^	2.5	0.009	64.0 ^a^	0.8	57.4 ^b^	2.0	81.0 ^c^	2.0	<0.0001
Iron (100)	82.9	0.8	94.4	0.7	<0.0001	92.2 ^a^	0.6	96.1 ^b^	0.6	92.9 ^ab^	1.2	94.6 ^ab^	0.9	94.5 ^ab^	1.1	0.001	95.4 ^a^	0.4	89.2 ^b^	1.5	98.6 ^c^	0.5	<0.0001
Potassium (100)	85.7	0.7	85.9	1.0	0.84	84.7 ^a^	0.8	85.0 ^a^	1.0	79.1 ^ab^	2.4	71.5 ^b^	2.3	84.1 ^a^	1.8	<0.0001	89.6 ^a^	0.5	86.5 ^a^	1.4	97.2 ^b^	0.9	<0.0001
Magnesium (100)	92.5	0.5	92.7	0.7	0.81	89.5	0.7	89.2	0.9	86.5	1.6	90.8	1.3	91.4	1.4	0.27	93.4 ^a^	0.4	94.4 ^ab^	0.9	97.9 ^b^	0.7	0.002
Added sugars (---)	141.8	10.2	46.1	5.1	<0.0001	66.5 ^a^	7.7	22.8 ^b^	3.6	27.3 ^ab^	6.8	26.6 ^ab^	6.6	45.6 ^ab^	10.5	0.0001	28.9 ^a^	3.1	8.0 ^b^	2.0	20.4 ^ab^	5.1	0.03
Saturated fats (---)	63.9	2.5	28.1	2.5	<0.0001	23.6	1.6	20.7	2.2	22.7	4.0	17.9	3.2	16.9	2.9	0.39	27.2 ^a^	1.3	17.8 ^b^	3.2	41.8 ^c^	5.3	<0.0001
Sodium (---)	55.1	3.0	97.3	4.9	<0.0001	97.3 ^a^	4.6	85.4 ^a^	5.0	228.9 ^b^	15.9	168.1 ^c^	9.5	91.9 ^a^	7.8	<0.0001	113.5 ^a^	3.4	71.8 ^b^	5.8	117.9 ^a^	9.7	<0.0001

HEI-2015, Healthy Eating Index 2015; NRF9.3, Nutrient-Rich Food Index 9.3. ^1^ Each cluster represents a group of meals with a similar dish-based dietary pattern. For breakfast, differences between two dietary patterns were tested with an independent t-test. For lunch and dinner, mean values between clusters without a common letter differ, *p* < 0.05 tested with ANOVA and Tukey post-hoc test. ^2^ Calculated as the sum of all components scores. A maximum score is shown in parenthesis. A higher score indicates a higher diet quality. ^3^ Defined as the ratio of the sum of polyunsaturated and monounsaturated fatty acids to saturated fatty acids. ^4^ Calculated as the sum of scores for nine nutrients to encourage (i.e., protein, dietary fibre, vitamins A, C and D, calcium, iron, potassium, and magnesium) minus the sum of scores for three nutrients to limit (i.e., added sugars, saturated fats, and sodium). A maximum score is shown in parenthesis. For added sugars, saturated fats, and sodium components, a maximum score is infinite depending on the amount. A higher score indicates a higher diet quality, except for added sugars, saturated fats, and sodium components, for which a higher score indicates a lower diet quality.

## Data Availability

The data presented in this study are available on request from the corresponding author. The data are not publicly available due to privacy or ethical restrictions.

## Supplementary Materials

The following are available online at https://www.mdpi.com/2072-6643/13/1/67/s1, Table S1: Dish groups used in this study, Table S2: Reference daily values used to calculate NRF9.3, Table S3: Food groups used in this study, Table S4: Comparison of weight (g) of food groups among dish-based dietary patterns for each meal.
