# Supplementary material for: Identification of Dish-Based Dietary Patterns for Breakfast, Lunch, and Dinner and Their Diet Quality in Japanese Adults
Source: Nutrients. 2020 Dec 28;13(1):67. doi: 10.3390/nu13010067 (PMC7824520; doi:10.3390/nu13010067)
Supplement: Supplementary file 1 [file nutrients-13-00067-s001.pdf]

Table S1. Dish groups used in this study

Dish group		
**ID**	**Name**	**Dish items**
1	Rice	White rice, brown rice, rice with mixed grains; red rice, beans rice; takikomi or mixed rice (rice seasoned and cooked with various ingredients), boiled rice with natto, raw egg mixed with white rice
2	Porridge	Porridge, oatmeal; ochazuke (boiled rice with tea); zousui (porridge of rice and vegetables), risotto, gukbap (Korean rice soup)
3	Curry and rice/omelette rice	Curry and rice, hash and rice; omelette rice
4	Fried rice/pilaf/doria	Pilaf, chicken rice, dry curried rice; doria, rice gratin; fried rice; gratin, lasagne
5	Sushi/rice bowl dishes	Nigiri sushi, rolled sushi; chirashizushi; kaisen-don (bowl of rice topped with sashimi); beef rice bowl; pork cutlet rice bowl; oyako-don (chicken and egg rice bowl), oyakodon-like dish made with something other than chicken; grilled eel and rice; other bowls
6	Rice ball	Rice ball
7	Rice cake	Rice cake (not sweet)
8	Plain bread	Toast; rolls; coppepan (similar to a hot dog bun); French bread; Danish pastry, croissant
9	Sweet bread/savoury bread	Sweet bread; toast with cheese, tuna, ham or other toppings; French toast, garlic toast; filled & stuffed bread; hot dog, corn dog; sandwich; hamburger
10	Udon/soba	Udon (thick wheat noodles), houtou, suiton (flour dumplings boiled in soup); somen, hiyamugi, nyuumen (thin wheat noodles); soba (buckwheat noodles); yakisoba (fried noodles); yakiudon (stir-fried udon);
11	Pasta	Pasta
12	Ramen	Instant noodle; other ramen, champon (a dish of noodles with seafood, vegetables, etc.)
13	Other noodles	Somin chanpuru (Okinawan-style stir-fried somen noodles); hiyashi-chuka (chilled Chinese noodles); fried rice noodles; Okinawa soba, soki soba; other noodles
14	Okonomiyaki/takoyaki/pizza	Okonomiyaki (savoury pancake with various ingredients); takoyaki (octopus dumplings), negiyaki (green spring onion pancake); pizza; tacos; sweet pancakes
15	Cereal	Cereal
16	Potato dishes	Nikujaga (meat and potato stew); simmered potatoes; steamed, boiled, or roasted potatoes; grated yam; yam strips; potato salad; potato croquette; cream croquette; German potato; cheese-grilled potato; stir-fried potato; fried potato, potato tempura
17	Natto (fermented soybeans)	Natto (fermented soybeans)

Table S1. *Cont.*

Dish group		
**ID**	**Name**	**Dish items**
18	Tofu dishes	Chilled tofu; boiled tofu; agedashi-tofu (deep-fried tofu); unohana (simmered soybean pulp); iri-doufu (stir-fried tofu), simmered tofu; koya-doufu (freeze-dried tofu simmered in seasoned broth); mabo tofu, tofu topped with a sticky sauce; soybean hamburg, tofu hamburg; tofu dengaku (tofu with sweet miso sauce), tofu steak, grilled tofu; fried tofu, fried atsuage (thick fried tofu); raw or baked atsuage; simmered atsuage, simmered ganmodoki (fried mixture of crushed tofu, vegetables and ground yam); grilled aburaage (thin fried tofu); yuba (film formed on surface of warmed soy milk);
19	Bean dishes	Boiled beans (soybean, black soybean); boiled beans (except for soybeans); boiled beans; kinako (roasted and ground beans), roasted soybean
20	Nut dishes	Ginkgo nuts, chestnuts; goma-tofu, peanut tofu (tofu-like gel made from sesame or peanut paste and kudzu starch); other nuts, sesame seeds
21	Japanese-style vegetable side dishes	Aemono (sauce-dressed dishes), nuta (salad with vinegar and miso); ohitashi (boiled vegetables); sunomono (vinegared dish); shiomomi, misotsuke (vegetables seasoned with salt or miso)
22	Raw or boiled vegetable dishes	Grated daikon radish; tomato; other raw vegetables, vegetable garnish; boiled, steamed, or canned vegetables; vegetable salad; seaweed salad; harusame noodle salad; spaghetti salad, macaroni salad; boiled corn
23	Simmered vegetable dishes	Simmered dried-radish; kinpira; furofuki daikon (simmered daikon radish with miso sauce); yasai dengaku (vegetables with sweet miso sauce); simmered vegetables (root vegetables); simmered vegetables (other vegetables); vegetables topped with a sticky sauce
24	Grilled or stir-fried vegetable dishes	Grilled eggplant; other grilled vegetables; mabo eggplant, stir-fried eggplant; papaya stir fry; goya champuru (bitter melon stir fry with tofu and egg); vegetable stir fry (others); vegetable stir fry (vegetables only); harusame noodle stir fry
25	Deep-fried vegetable dishes	Kakiage (mixed tempura); tempura and deep-fried vegetables; deep-fried stuffed vegetables
26	Mushrooms/seaweed/konnyaku dishes	Mozuku, mekabu (root of wakame seaweed); wakame, kelp; laver; stewed mushrooms; stewed seaweed, tangle roll; konnyaku (paste made from konnyaku flour) dish; stir-fried or grilled mushrooms
27	Fruit/fruit dishes	Banana; strawberry (raw); apple (raw); persimmon (raw); pear (raw); grape (raw); mandarin orange (raw); other citrus (raw); other raw fruits; assorted fruit platter; canned fruits, compote; dried persimmon; other dried fruits; fruit punch; fruit salad
28	Raw fish dishes	Sashimi (fish); sashimi (squid, shrimp, octopus, shellfish); sashimi (assorted seafood); yamakake (raw fish topped with grated yam); marinated or picked seafood
29	Seafood dishes (steamed/boiled/ simmered)	Boiled seafood; steamed seafood; buri daikon (yellowtail and daikon cooked with soy sauce); fry-simmered fish, nanbanzuke (fried fish in sweet and peppery vegetable sauce); simmered fish with seasoning (white fish including salmon); simmered fish with seasoning (other fish); simmered squid, shrimp, octopus, or shellfish

Table S1. *Cont.*

Dish group		Dish items
**ID**	**Name**	
30	Grilled or stir-fried seafood dishes	Kasuzuke (fish pickled in sake lees), miso-marinated fish; kabayaki (broiled fish); teriyaki fish, yawatamaki (burdock rolled with fish); meuniere fish, fish in butter sauce; dried fish (white fish); dried fish (other fish); grilled fish (salmon and trout); grilled fish (white salted fish except salmon or trout); grilled fish (other fish); grilled squid, shrimp, octopus, or shellfish; happousai (chop suey); shrimps with chili sauce; other fried fish and shellfish
31	Deep-fried fish dishes	Fish tempura, fried fish (white fish including salmon); fish tempura, fried fish (other fish); shrimp tempura, fried shrimp (excluding kara-age); squid tempura, fried squid (excluding kara-age); shrimp or squid kara-age; fried oyster; fried crab, octopus or scallop; fish topped with a sticky sauce
32	Fish jelly product dishes	Fish jelly products (raw, baked); simmered dish containing fish jelly products; stir-fried food containing fish jelly products; fish jelly products tempura and fried fish jelly products
33	Canned fish/seafood delicacy	Salmon flakes; canned tuna; canned Pacific saury, mackerel or sardines; small dried sardines; dried whitebait; smoked squid; cod roe; salmon roe; other fish eggs; salted fish and shellfish, fish delicacy
34	Meat dishes (raw/boiled/steamed)	Raw meat, roast beef; tonsoku (pig's feet), boiled pork; steamed chicken
35	Gyoza dumplings/shumai/meatballs	Fried gyoza dumplings; boiled or steamed gyoza dumplings (except shrimp dumplings); deep-fried gyoza dumplings; shumai (steamed Chinese dumplings); meat-stuffed vegetables; meat roll-ups; meatballs (beef, pork); seasoned ground meat, meat sauce; ground beef
36	Simmered meat with seasoning	Simmered beef; simmered pork; boiled dish (Okinawa style); chikuzenni (simmered root vegetables with chicken); other simmered chicken dish; simmered lever or tripe; stuffed cabbage rolls
37	Grilled or stir-fried meat dishes	Spit-roasted beef or pork; beef steak; other grilled beef; chicken steak, chicken piccata; ginger fried pork; other grilled pork; other pork dishes; chicken meatballs; yakitori (skewered chicken); teriyaki chicken; other grilled chicken; teppanyaki (food roasted on a hot plate.), barbeque; grilled horsemeat or mutton; meatloaf; stir-fried beef; stir-fried pork; stir-fried chicken; stir-fried or grilled lever, tripe
38	Deep-fried meat dishes	Breaded ground meat cutlet; subuta (sweet and sour pork); chicken namban (fried chicken with vinegar and tartar sauce); pork cutlet; pork kara-age; chicken kara-age; kushikatsu (deep-fried skewers) and other deep-fried meat
39	Processed meat dishes	Wieners, sausage (not deep-fried); ham; ham roll-ups; bacon; barbecued pork; stir-fried processed meat (except for wieners, sausage); deep-fried processed meat
40	Egg dishes	Boiled egg, soft-boiled egg, raw egg; fried egg; ham and egg, bacon and egg; tamago-yaki (Japanese rolled omelette); omelette; scrambled egg; niratama (garlic chive scrambled eggs); egg tofu, chawanmushi (steamed egg custard); tamagotoji (egg soup); kanitama (crab and egg); other egg dishes

Table S1. *Cont.*

Dish group		Dish items
ID	Name	
41	Milk/soymilk	Milk (normal or high fat); milk (low fat); soymilk
42	Yogurt/cheese	Yogurt; fruit yogurt; cheese
43	Japanese confectionery	Daigakuimo (candied sweet potato), sweet potato (confection made from sweet potatoes); kinton (mashed sweet potatoes), sweet potato ball; dried sweet potato; rice cracker, arare (glutinous rice cracker), other savoury rice crackers; rakugan (moulded dry confectionary made from fine roasted glutinous rice flour and sugar), okoshi (puffed sweet rice cake), boro (small balls of baked starch dough); daifuku (sweet rice cake stuffed with red bean paste), rice-cake sweets; manjuu (sponge cake stuffed with red bean paste), monaka (glutinous rice wafers with red bean paste filling); ohagi (rice ball coated with sweetened red beans, soybean flour, or sesame and salt), shiruko (sweet red-bean soup); taiyaki (a fish-shaped pancake filled with bean jam), dorayaki (Japanese pancake sandwich with red bean paste filling), (Japanese waffle stuffed with red bean paste); castella; yokan (sweet beans jelly), nerikiri (Japanese artistic cake); other beans or nut sweets; karinto (crunchy deep fried wheat flour dough coated with white sugar), imokenpi (deep fried sweet potato chips, coated with sugar syrup), vegetable chips; steamed bread, chukaman (Chinese steamed bun); raw sugar block; other Japanese confectionery
44	Ice cream/jelly/pudding/cake	Shaved ice, sherbet; ice cream, milk shake, pudding, bavaroa, mousse; jelly, agar; waffle, cream puff; doughnut; cake and other Western confectionery
45	Chocolate/cookies/candy	Cookie, cracker, pretzel, wafer; chocolate, chocolate confectionery, candy, gum, marshmallow
46	Snacks	Kakinotane (seed-sized rice crackers); potato crisps; snack food; assortment of snacks
47	Coffee/tea	Coffee (excluding coffee-flavoured milk); tea
48	Japanese and Chinese tea	Other tea (barley tea, oolong tea etc.); water, sugar-free carbonated water, 0 kcal beverage
49	Cocoa/milk beverage	Cocoa, Milo; coffee-or fruit- flavoured milk, fruit milk, latte; lactic acid bacteria beverage, yogurt drink
50	Vegetable and fruit juice	Vegetable juice, green juice; fruit juice
51	Soft drink	Sports drink; sugar-sweetened soda; other soft drink; kudzu-yu (arrowroot gruel), amazake (sweet sake); energy drink; shougayu (ginger soup), tamagoyu (egg drink); vinegar drink
52	Japanese sake/shochu	Japanese sake, shochu, awamori (on the rocks/with tea, water, hot water); white sake
53	Beer	Beer, happoushu (sparking alcoholic beverages)
54	Fruit liquor/wine	Fruit liquor, cocktail; wine; whiskey and other Western liquors

Table S1. *Cont.*

Dish group		
**ID**	**Name**	**Dish items**
55	Miso soup	Miso soup; suimono and sumashijiru (clear soup); other kinds of Japanese style soup; tonjiru (miso soup with pork and vegetables); nakami-jiru (Okinawan pork tripe soup); kasujiru (soup with a sake lees base); somen soup; soki-jiru (Okinawan pork spareribs soup); zouni (rice cakes boiled with vegetables)
56	Western or Chinese soup	Potage, cream soup; wan-tun soup, dumpling soup, harusame soup; other Western or Chinese soup
57	Hot pot dishes	Borscht, pot-au-feu; stew, beef stroganoff; oden (fish paste stew); shabu-shabu (thin-sliced meat simmered in hot water); sukiyaki hot pot; other hot pot dishes
58	Tsukudani/pickles	Tsukudani (seafood); tsukudani (vegetables and seaweeds); pickled plum; kimchi; pickled scallion, vinegar pickles; other pickles; miso
59	Spring rolls/assorted fried foods	Deep-fried food wrapped in dumplings skins; spring roll; assorted fried foods

Table S2. Reference daily values used to calculate NRF9.3 [a]

Variable	Values for men aged 30–49 years
Energy (kcal) [b]	2700
Energy (kJ) [b]	11297
Qualifying nutrients	
Protein (g) [c]	65
Dietary fibre (g) [d]	21
Vitamin A (μg RAE) [c]	900
Vitamin C (mg) [c]	100
Vitamin D (μg) [e]	8.5
Calcium (mg) [c]	750
Iron (mg) [c]	7.5
Potassium (mg) [d]	3000
Magnesium (mg) [c]	370
Disqualifying nutrients	
Added sugars (g) [f]	33.75
Saturated fats (g) [g]	21
Sodium (g NaCl equivalent) [dh]	7.5

NRF9.3, Nutrient-Rich Food Index 9.3; RAE, retinol activity equivalent. [a] Values were derived from the Dietary Reference Intakes for Japanese, 2020 [32], except for added sugars (as shown below). [b] Estimated Energy Requirement for moderate level of physical activity. [c] Recommended Dietary Allowance. [d] Tentative dietary goal for preventing lifestyle-related diseases (DG). [e] Adequate Intake. [f] Determined based on the World Health Organization's conditional recommendation (5% of energy) [34]. [g] Determined based on the DG value (7% of energy). [h] 7.5 g NaCl equivalent = 2952 mg sodium.

Table S3. Food groups used in this study

Food groups	Major food items
Rice	White rice, wheat, millet, amaranthus, corn
Bread	White bread, butter roll, baguette, croissant
Noodles	Spaghetti, Japanese noodles (buckwheat and Japanese wheat noodles), instant noodles, Chinese noodles
Other grain products	Wheat flour, wheat products, cornflakes
Potatoes	Potatoes, sweet potatoes, yams and taro; konnyaku
Sugars	Jam and marmalade, sugar, honey, maple syrup
Total vegetables	Carrots, onions, cabbage, Japanese leek, cucumbers, pumpkins, tomatoes, green peppers, broccoli, lettuce, Chinese cabbage, bean sprouts, radishes, cauliflower, eggplants, burdock, lotus root, mushrooms, wakame, hijiki, laver
Pulses (including nuts)	Peanuts, other nuts, tofu, other tofu products, natto, beans, miso
Fruits	Raisins, canned fruits, oranges, bananas, apples, strawberries, grapes, peaches, pears, persimmons, kiwi fruits, melons, watermelons
Fish and shellfish	Skipjack tuna, fish products, salmon, squid, sardine, shrimp, Pacific saury, shell fish
Meats	Pork, chicken, beef, sausage, ham, ground meet, bacon
Eggs	Eggs
Dairy products	Milk, cheese, yogurt
Fats and oils	Lard, butter, margarine, mayonnaise, salad dressing, oil
Confectioneries	Japanese bread with a sweet filling, doughnuts, pancakes, potato chips, rice crackers, Japanese sweets, cakes, cookies and biscuits, chocolates, candies, caramels, and chewing gum, jellies, ice cream
Fruit and vegetable juice	Fruit juice, vegetable juice
Alcoholic beverages	Beer, sake, shochu, whiskey, wine
Soft drinks	Sugar-sweetened beverages
Tea and coffee	Green tea, coffee, barley tea, black tea, oolong tea, brown rice tea
Seasonings and spices	Soy sauce, salt, vinegar, soup stock, ketchup, herb and spice

Table S4. Comparison of weight (g) of food groups among dish-based dietary patterns for each meal [1]

	Breakfast					Lunch											Dinner						
	Bread-based (n = 983)		Rice-based (n = 479)			Bread (n = 781)		Rice-based (n = 393)		Ramen (n = 99)		Udon/soba (n =125)		Sushi/rice bowl dishes (n = 106)			Miscellaneous (n = 1211)		Meat dish and Beer (n = 165)		Hot pot dishes (n = 124)		
	**Mean**	**SE**	**Mean**	**SE**	***P***	**Mean**	**SE**	**Mean**	**SE**	**Mean**	**SE**	**Mean**	**SE**	**Mean**	**SE**	***P***	**Mean**	**SE**	**Mean**	**SE**	**Mean**	**SE**	***P***
Rice	38.0	2.3	117.3	4.3	<0.0001	134.8 [a]	3.5	168.6 [b]	4.3	40.8 [c]	7.3	54.2 [c]	7.4	208.1 [d]	7.6	<0.0001	143.8 [a]	3.0	97.6 [b]	7.7	113.8 [b]	9.6	<0.0001
Bread	37.1	1.3	15.1	1.6	<0.0001	11.7 [a]	1.1	3.6 [b]	0.9	2.5 [b]	1.4	2.2 [b]	1.1	0.8 [b]	0.6	<0.0001	2.0	0.4	0.9	0.6	2.3	1.1	0.57
Noodles	2.3	0.7	6.3	1.5	0.006	13.6 [a]	1.7	18.1 [a]	2.8	200.5 [b]	6.9	221.2 [c]	7.2	11.7 [a]	4.3	<0.0001	32.4	2.4	22.6	4.7	23.9	6.3	0.22
Other grain products	1.7	0.3	3.1	0.9	0.07	6.4	1.0	4.8	0.8	1.9	0.9	4.3	0.9	6.3	1.6	0.35	5.8	0.5	8.8	1.8	5.5	2.7	0.19
Potatoes	2.3	0.3	5.0	0.8	0.0002	15.3 [a]	1.0	15.5 [a]	1.7	2.1 [b]	0.7	4.1 [b]	1.2	8.2 [ab]	1.8	<0.0001	20.4 [a]	1.1	25.9 [a]	4.2	45.9 [b]	6.1	<0.0001
Sugar	4.3	0.3	2.9	0.4	0.007	4.1 [a]	0.3	3.6 [a]	0.3	1.9 [a]	0.4	2.5 [a]	0.4	8.9 [b]	1.9	<0.0001	3.4	0.2	3.6	0.5	2.8	0.4	0.53
Pulses and nuts	10.4	1.1	30.2	2.0	<0.0001	15.2	1.2	14.0	1.6	9.3	3.5	7.0	1.5	16.7	2.6	0.03	32.2 [a]	1.6	26.4 [a]	3.9	69.1 [b]	6.6	<0.0001
Total vegetables	23.2	1.3	46.8	2.4	<0.0001	88.8 [a]	2.9	92.2 [a]	3.9	61.6 [b]	6.4	67.0 [b]	6.1	87.3 [ab]	6.2	0.0003	129.7 [a]	2.9	146.2 [a]	8.3	247.9 [b]	12.3	<0.0001
Fruit	24.2	1.5	20.0	2.0	0.11	16.9	1.2	13.9	1.6	8.8	2.4	10.9	2.5	16.2	3.5	0.07	12.1	1.1	15.5	3.6	10.0	2.7	0.42
Fish and shellfish	4.5	0.5	10.0	1.1	<0.0001	20.3 [ab]	1.3	24.7 [a]	1.8	7.0 [c]	1.5	12.2 [bc]	1.6	41.2 [d]	6.9	<0.0001	39.6 [a]	1.6	52.1 [b]	4.6	38.7 [ab]	5.7	0.03
Meat	6.3	0.5	9.0	1.0	0.01	29.6 [a]	1.3	36.5 [b]	2.2	20.9 [ac]	2.7	17.3 [c]	2.3	44.0 [b]	4.3	<0.0001	50.0 [a]	1.7	61.6 [a]	5.2	82.8 [b]	6.1	<0.0001
Eggs	9.7	0.7	17.9	1.3	<0.0001	12.6 [a]	0.8	17.5 [bc]	1.3	11.5 [ab]	2.1	11.9 [ab]	1.8	24.4 [c]	2.4	<0.0001	12.8	0.7	13.5	1.9	14.8	2.4	0.66
Dairy products	66.2	2.9	41.9	3.5	<0.0001	19.4 [a]	1.8	10.2 [b]	1.7	6.9 [b]	3.3	7.7 [b]	2.5	9.2 [ab]	2.8	0.0001	14.7	1.4	10.2	2.7	11.8	2.7	0.42
Fat and oil	4.7	0.2	3.5	0.4	0.003	8.9	0.5	8.2	0.8	7.3	0.7	7.1	0.9	5.8	0.9	0.21	8.5 [a]	0.4	10.0 [a]	1.2	4.0 [b]	0.7	0.0017
Confectioneries	7.1	0.8	2.3	0.7	0.0002	6.4	0.9	4.7	1.2	9.4	3.0	3.8	1.8	7.2	2.0	0.34	4.1	0.6	3.4	0.9	3.0	1.3	0.75
Fruit and vegetable jui	7.9	1.2	6.1	1.5	0.37	5.9	1.5	1.4	0.7	3.0	1.9	9.4	5.5	1.9	1.9	0.15	2.5	0.6	2.8	1.5	0.0	0.0	0.41
Alcoholic beverages	0.2	0.0	0.7	0.1	<0.0001	4.6	1.1	2.7	0.5	5.4	3.8	5.5	4.2	9.5	3.7	0.33	22.9 [a]	2.2	673.3 [b]	25.1	97.5 [c]	16.8	<0.0001
Soft drinks	5.1	1.1	3.5	0.9	0.34	8.9	2.0	1.9	1.3	2.9	1.6	4.0	1.8	0.0	0.0	0.052	10.6	1.9	8.3	4.2	17.5	7.9	0.48
Tea and coffee	173.6	6.1	227.4	7.8	<0.0001	121.5 [a]	5.7	296.7 [b]	8.9	146.7 [a]	17.4	151.0 [a]	13.4	156.4 [a]	14.2	<0.0001	150.9 [a]	5.0	51.3 [b]	8.0	152.2 [a]	16.2	<0.0001
Seasonings and spices	8.2	1.0	19.4	2.2	<0.0001	40.7 [a]	2.4	38.7 [a]	3.2	121.6 [b]	12.6	78.0 [c]	8.5	67.7 [c]	7.7	<0.0001	44.8 [a]	2.0	43.0 [a]	4.3	68.9 [b]	9.3	0.001

SE, standard error. [1] Each cluster represents a group of meals with a similar dish-based dietary pattern. For breakfast, differences between two dietary patterns were tested with an independent t-test. For lunch and dinner, mean values between clusters without common letter differ, $P < 0.05$ tested with ANOVA and Tukey post-hoc test.
